# Older Adults’ Knowledge of Geriatric Depression and Its Related Factors

**DOI:** 10.1192/j.eurpsy.2024.570

**Published:** 2024-08-27

**Authors:** Y.-F. Tsai, S.-H. Lee

**Affiliations:** ^1^School of Nursing, Chang Gung University; ^2^Department of Psychiatry, Chang Gung Memorial Hospital at Linkou, Tao-Yuan , Taiwan, Province of China

## Abstract

**Introduction:**

Even though depression is a severe health issue among older adults, few studies have explored their knowledge of geriatric depression.

**Objectives:**

This study aimed to explore older adults’ knowledge of geriatric depression and its related factors.

**Methods:**

A cross-sectional survey was conducted. Older adults were recruited by convenience from outpatient clinics of three hospitals in Taiwan.

**Results:**

A total of 327 older adults participated in this study. Their mean score of knowledge was 7.73 (SD=2.12, Range=2-12) on an 18-item knowledge scale, indicating poor knowledge of geriatric depression. Females had significantly higher geriatric depression knowledge scores than males (t=2.50, p=0.01). Junior and senior high school graduates had significantly higher geriatric depression knowledge scores than illiterate and primary school graduates (F=10.23, p<0.01). In addition, their geriatric depression knowledge scores also differed by religious belief (F=4.91, p<0.01), living status (F=8.64, p<0.01), and perceived health condition (F=8.81, p<0.01). Buddhists had significantly higher geriatric depression knowledge scores than Taoists. Living with partners and perceiving their health status as fair and good tended to have higher geriatric knowledge scores than their counterparts. However, their geriatric depression knowledge scores did not significantly correlate with their mean scores of social distance toward older adults with depression.

**Conclusions:**

Older adults tended to have poor geriatric depression knowledge. Improving their knowledge shall be an urgent task. Our results may serve as references for developing further depression prevention.

**Disclosure of Interest:**

None Declared

